# Effects of caffeine intake on depressive and anxiety symptoms: a systematic review and meta-analysis of randomized controlled trials

**DOI:** 10.3389/fnut.2026.1950050

**Published:** 2026-10-29

**Authors:** Ailing Zhang, Yudan Ni, Zhongyu Zhang, Jun Wang, Weiwei Chen, Furong Chang, Hengyu Cui, Changzheng Yuan, Dong Hang, Lin Li

**Affiliations:** 1 Department of Nuclear Medicine, Nanjing First Hospital, Nanjing Medical University, Nanjing, China; 2 Jingjiang College, Jiangsu University, Zhenjiang, China; 3 Health Management Center, Nanjing First Hospital, Nanjing Medical University, Nanjing, China; 4 Department of Epidemiology, School of Public Health, Changzhou Medical Center, Gusu School, Nanjing Medical University, Nanjing, China; 5 Center of Clinical Big Data and Analytics of the Second Affiliated Hospital and School of Public Health, Zhejiang University School of Medicine, Hangzhou, Zhejiang, China; 6 Department of Social Services, Nanjing First Hospital, Nanjing Medical University, Nanjing, China

**Keywords:** anxiety symptoms, caffeine intake, depressive symptoms, dose stratification, meta-analysis, randomized controlled trials

## Abstract

**Background:**

Caffeine is one of the most widely consumed psychoactive dietary components worldwide. However, its effects on affective symptoms remain uncertain, with inconsistent findings regarding depressive and anxiety outcomes. This systematic review and meta-analysis aimed to evaluate the effects of quantified caffeine intake on affective symptoms based exclusively on randomized controlled trial (RCT) evidence.

**Methods:**

The review followed the Preferred Reporting Items for Systematic Reviews and Meta-Analyses (PRISMA) 2020 statement and was registered in the International Prospective Register of Systematic Reviews (PROSPERO; CRD42024564399). PubMed/MEDLINE, Embase, the Cochrane Library, Web of Science, CBM, VIP, CNKI, and Wanfang Data were searched from inception to May 16, 2026. Random-effects meta-analyses used restricted maximum likelihood estimation with Hartung–Knapp adjustment. Standardized mean differences (SMDs) with 95% confidence intervals (CIs) and, where appropriate, 95% prediction intervals (PIs) were reported. Clinical coherence was examined according to exposure pattern and psychiatric status, and dose-stratified analyses were exploratory.

**Results:**

Fifteen RCTs involving 668 participants contributing to the selected comparisons were included. For depressive symptoms, the broad pooled estimate did not show clear evidence of a difference between caffeine and control conditions [SMD = −0.49, 95% CI: (−1.33, 0.34); *I*^2^ = 92.89%; 95% PI: (−2.87, 1.88)]. Anxiety scores were higher on average after caffeine exposure [SMD = 0.51, 95% CI: (0.12, 0.89); *I*^2^ = 90.30%], although the 95% PI crossed the null (−0.95, 1.96). In acute non-psychiatric comparisons, pooled estimates were −0.15 [95% CI: (−2.12, 1.81)] for depressive symptoms and 0.38 [95% CI: (−0.02, 0.79)] for anxiety. Exploratory dose stratification showed higher anxiety scores in the >200 mg category [SMD = 0.97, 95% CI: (0.55, 1.39)] but not in the ≤200 mg category [SMD = −0.05, 95% CI: (−0.41, 0.30)]. Certainty of evidence was very low for both outcomes, and small-study effects were detected for anxiety.

**Discussion:**

Randomized evidence does not support a uniform effect of caffeine across settings. The positive broad anxiety estimate should be interpreted cautiously because of substantial heterogeneity, a prediction interval spanning the null, and very-low-certainty evidence. Dose-category findings remain exploratory and do not establish a clinical threshold or continuous dose–response relationship.

**Systematic Review Registration:**

https://www.crd.york.ac.uk/prospero/display_record.php?RecordID=564399, PROSPERO CRD42024564399.

## 1 Introduction

Depressive and anxiety disorders are major contributors to the global burden of disease, affecting an estimated 280 million and 301 million people worldwide, respectively (1). The COVID-19 pandemic has further intensified this burden, underscoring the need to identify modifiable lifestyle and dietary factors that may contribute to the prevention and management of mood-related symptoms, particularly given the variable effectiveness and tolerability of conventional pharmacological treatments.

Among modifiable dietary exposures, coffee is one of the most widely consumed beverages worldwide and has well-characterized behavioral and physiological effects (2, 3). These effects are largely attributable to caffeine (1,3,7-trimethylxanthine), the principal psychostimulant constituent of coffee (4, 5). Although coffee contains numerous other bioactive compounds, caffeine is considered the predominant mediator of its acute effects on affective and central nervous system function (4, 5). Following ingestion, caffeine is rapidly absorbed from the gastrointestinal tract, with peak plasma concentrations typically reached within 30–60 min (6). At customary dietary doses, its psychotropic effects are mediated primarily by non-selective, competitive antagonism of central adenosine A_1_ and A_2A_ receptors (7).

The relationship between caffeine intake and affective regulation appears bidirectional and mechanistically complex. Antagonism of central adenosine receptors increases cerebral energy metabolism while reducing cerebral blood flow; together with enhanced noradrenergic activity and region-specific dopaminergic modulation, these effects may contribute to anxiety-related responses (8, 9). By contrast, caffeine may transiently attenuate depressive symptoms through enhanced serotonergic and dopaminergic neurotransmission (10). Because caffeine is consumed or administered through diverse dietary and experimental preparations, quantifying exposure according to the administered caffeine dose provides a common metric for comparing affective outcomes across studies, although differences in formulation and co-occurring bioactive constituents may contribute to between-study heterogeneity.

Despite these biologically plausible pathways, epidemiological evidence remains inconsistent. Previous meta-analyses of observational studies have reported inverse associations between overall caffeine intake and depressive symptoms (11, 12). However, these analyses were often limited by restricted population characteristics and could not distinguish dose-specific effects from the broader influence of correlated dietary and lifestyle factors. A recent meta-analysis similarly linked higher caffeine intake to an increased risk of anxiety, but its conclusions were derived predominantly from observational data (13). The susceptibility of cross-sectional and prospective cohort studies to residual confounding and exposure misclassification therefore limits causal inference regarding the effects of caffeine on affective outcomes.

## 2 Methods

This systematic review and meta-analysis was conducted according to a prespecified protocol and reported in accordance with the Preferred Reporting Items for Systematic Reviews and Meta-Analyses (PRISMA) 2020 statement (14). The protocol was prospectively registered in the International Prospective Register of Systematic Reviews (PROSPERO; registration number CRD42024564399; https://www.crd.york.ac.uk/prospero/display_record.php?RecordID=564399).

### 2.1 Study identification and selection

Eligibility criteria were defined *a priori* according to the Population, Intervention, Comparator, Outcome, and Study design (PICOS) framework (15). Eligible studies were randomized controlled trials using parallel-group or crossover designs that compared quantified caffeine exposure, administered either as isolated caffeine or through a caffeine-containing food or beverage, with placebo, decaffeinated, or caffeine-free control conditions and assessed depressive or anxiety symptoms using prespecified symptom measures.

To systematically identify relevant studies, a comprehensive literature search was conducted in four English-language databases (PubMed/MEDLINE, Embase, the Cochrane Library, and Web of Science) and four Chinese-language databases [Chinese Biomedical Literature Database (CBM), VIP Database for Chinese Technical Periodicals (VIP), China National Knowledge Infrastructure (CNKI), and Wanfang Data]. All databases were searched from their inception to May 16, 2026. The search strategy combined controlled vocabulary [e.g., Medical Subject Headings (MeSH), where applicable] with free-text terms related to “coffee,” “caffeine,” “depression,” “anxiety,” and “randomized controlled trials”. To maximize retrieval of relevant studies, the reference lists of all included articles and relevant review papers were manually screened. Forward and backward citation tracking was also performed to identify additional eligible studies that might have been missed during the electronic database search.

Following removal of duplicate records, titles and abstracts were screened to identify potentially relevant studies. The full texts of potentially eligible articles were subsequently assessed against the prespecified eligibility criteria. Studies were included if they met all of the following criteria: (1) randomized controlled trials using either a parallel-group or crossover design; (2) administration of caffeine, either as isolated caffeine or through a caffeine-containing food or beverage, with a placebo, decaffeinated product, or otherwise caffeine-free control condition as the comparator; (3) assessment of depression or anxiety symptoms using a validated psychometric instrument; and (4) reporting of sufficient quantitative data to calculate an effect estimate and its corresponding variance, such as group means and standard deviations at baseline, post-intervention, or for changes from baseline, or other convertible statistical information. Studies were excluded if they: (1) were systematic reviews, meta-analyses, observational studies, animal studies, case reports, letters, editorials, or other non-primary research articles; (2) did not report a relevant depression or anxiety outcome; (3) represented duplicate publications or overlapping study populations, in which case the report containing the most complete or relevant data was retained; or (4) did not provide sufficient quantitative information for effect-size estimation. For studies that otherwise met the eligibility criteria but lacked the numerical information required to derive an effect estimate and its variance, we attempted to obtain the missing data from the corresponding authors. Studies were excluded only when the required information remained unavailable after these attempts.

Data were independently extracted by two investigators (AZ and YN) using a predefined form. Extracted information included study design, participant characteristics, sample size, intervention and comparator details, caffeine dose and delivery vehicle, exposure duration, washout period, outcome instrument, assessment time point, and quantitative outcome data. For studies with multiple intervention arms, doses, cohorts, or repeated assessments, all potentially eligible comparisons and time points were recorded before the explicit selection and handling procedures described below were applied. For crossover trials, paired data or paired effect estimates were used whenever available. Participant counts were reconciled at the study and cohort levels: participants in crossover or repeated-measure designs were counted once in the overall participant total, non-overlapping cohorts within the same publication were counted separately, and participants contributing to more than one outcome were not counted more than once in the overall review total. For multi-arm trials, only participants contributing to the selected analytical comparison were included in the corresponding synthesis-specific count. Caffeine intake was recorded in milligrams whenever an absolute dose could be established from the original report; weight-adjusted doses were otherwise retained in their originally reported units. Exploratory dose-classification procedures are detailed in Section 2.4 and further illustrated in Supplementary Table S1. The complete study- and comparison-level extraction dataset, including the numerical data used for effect-size estimation and participant reconciliation, is provided in Supplementary Table S2.

### 2.2 Risk of bias assessment

The risk of bias was assessed at the outcome level using the revised Cochrane risk-of-bias tool for randomized trials (RoB 2) (16), with depressive and anxiety outcomes evaluated separately. The standard RoB 2 tool was applied to parallel-group trials, whereas the crossover-specific version was used for crossover trials to address design-specific concerns, including potential period and carry-over effects. Judgments from both versions were summarized across the common domains of bias arising from the randomization process, deviations from intended interventions, missing outcome data, measurement of the outcome, and selection of the reported result. Each domain and the overall risk of bias were classified as “low risk,” “some concerns,” or “high risk.” Two investigators (AZ and YN) independently performed the assessments, with disagreements resolved through discussion or, when necessary, consultation with a third investigator (ZZ). Traffic-light plots and summary plots were generated using the robvis visualization tool (17).

### 2.3 Certainty assessment of evidence

The certainty of evidence for depressive and anxiety outcomes was assessed using the Grading of Recommendations Assessment, Development and Evaluation (GRADE) framework. Two investigators (AZ and YN) independently evaluated the evidence across five domains: risk of bias, inconsistency, indirectness, imprecision, and publication bias. Certainty was rated as high, moderate, low, or very low according to the GRADE criteria. Disagreements were resolved through discussion and consensus.

### 2.4 Statistical analysis

To avoid double-counting from multi-arm trials, shared control groups, and repeated assessments, only one eligible comparison and one assessment time point were included for each independent cohort and outcome, unless a publication contained genuinely independent cohorts or experiments. When the original investigators identified a primary or principal post-intervention assessment, that time point was selected. Otherwise, the latest assessment within the same acute experimental session was used, provided that it preceded any subsequent mood induction, cognitive task, exercise protocol, or other manipulation that could influence the outcome. Repeated measurements from the same participants were not treated as independent effect estimates. For trials with multiple eligible caffeine doses involving the same participants or a shared control group, only one dose comparison was retained; when no dose was designated as primary by the original study, the lower eligible dose was selected consistently for the primary pairwise analysis. When several eligible intervention arms in a parallel-group trial shared a common control and represented the same analytical contrast, intervention arms were combined where appropriate rather than reusing the control group. When multiple measures of the same outcome were available, preference was given to the measure most closely corresponding to the prespecified construct; for anxiety, validated multi-item measures of state anxiety were preferred to trait measures or single-item ratings when both were available. All eligible intervention arms, doses, assessment time points, selected comparisons, and reasons for selection are documented in Supplementary Table S3.

Random-effects meta-analyses were fitted using restricted maximum likelihood (REML) estimation of between-study variance, with the Hartung–Knapp adjustment applied to the 95% CIs of pooled estimates. Statistical heterogeneity was assessed using Cochran’s *Q* test and quantified using the *I*^2^ statistic and between-study variance (*τ*^2^) (18). For random-effects syntheses in which they could be estimated meaningfully, 95% prediction intervals (PIs) were calculated to characterize the range of true effects that might be expected across comparable study settings. Given the marked clinical heterogeneity in study populations, caffeine exposure patterns, and outcome constructs, the overall pooled estimates were interpreted as broad descriptive summaries rather than as common effects assumed to apply across settings. Clinical interpretation therefore relied primarily on the clinically stratified syntheses, while the broad estimates were retained to characterize the overall distribution of the randomized evidence rather than to support direct clinical inference. Clinical coherence was assessed with reference to exposure pattern and psychiatric status. Acute exposure was defined as single-session or single-day caffeine administration with outcome assessment conducted within the same experimental session, whereas sustained or repeated exposure referred to interventions administered over consecutive days or weeks. Psychiatric and non-psychiatric populations were evaluated separately where the available evidence allowed. Outcome measures were also appraised in relation to the constructs and assessment contexts they represented; statistical standardization alone was not considered sufficient to establish clinical equivalence between transient mood-state measures and clinical symptom scales. Within the clinically stratified analyses, quantitative synthesis was undertaken only when the contributing studies were judged to be sufficiently comparable; otherwise, findings were summarized narratively at the study level.

Exploratory subgroup analyses considered study design, caffeine dose, and outcome assessment approach where sufficient data were available. Differences according to exposure pattern and psychiatric status were addressed through the clinically stratified syntheses described above rather than treated as conventional subgroup analyses. Dose-stratified analyses used categories of ≤200 mg and >200 mg; the 200-mg cut point was used solely as an exploratory classification informed by caffeine-dose categories reported in previous literature (19) and was not regarded as an established clinical or biological threshold or as evidence of a continuous dose–response relationship. For weight-adjusted interventions, the primary exploratory dose category was assigned using the reported mg/kg dose together with the available study-level information. When the corresponding participant-level absolute dose could not be established unequivocally relative to the 200-mg boundary, the classification was designated as uncertain. These comparisons were retained in the primary exploratory dose-stratified analysis but excluded from the sensitivity analysis restricted to unambiguous dose classifications. Comparison-level dose assignments, including the handling of weight-adjusted and multiple-dose interventions, classification certainty, and inclusion in the restricted sensitivity analysis, are documented in Supplementary Table S1.

Additional sensitivity analyses examined restriction to isolated caffeine contrasts and sequential omission of individual comparisons. Robustness was judged from the stability of the direction and magnitude of the estimates rather than from statistical significance alone. Potential small-study effects and publication bias were explored using funnel plots, with regression-based tests for funnel-plot asymmetry undertaken only when the number of independent comparisons permitted meaningful assessment. All tests were two-sided, with *α* = 0.05.

## 3 Results

### 3.1 Study selection and characteristics of the included studies

The database search identified 1,139 records. After removal of 351 duplicates, 788 records underwent title and abstract screening, of which 735 were excluded. 53 reports were sought for retrieval, all of which were obtained and assessed for eligibility. 38 reports were subsequently excluded because of insufficient quantitative data for depressive or anxiety effect-size estimation (*n* = 23), absence of eligible depressive or anxiety outcomes (*n* = 13), or ineligible dietary supplement interventions (*n* = 2). 15 randomized controlled trials were included in the systematic review and meta-analysis. The study-selection process is shown in Figure 1.

**Figure 1:**
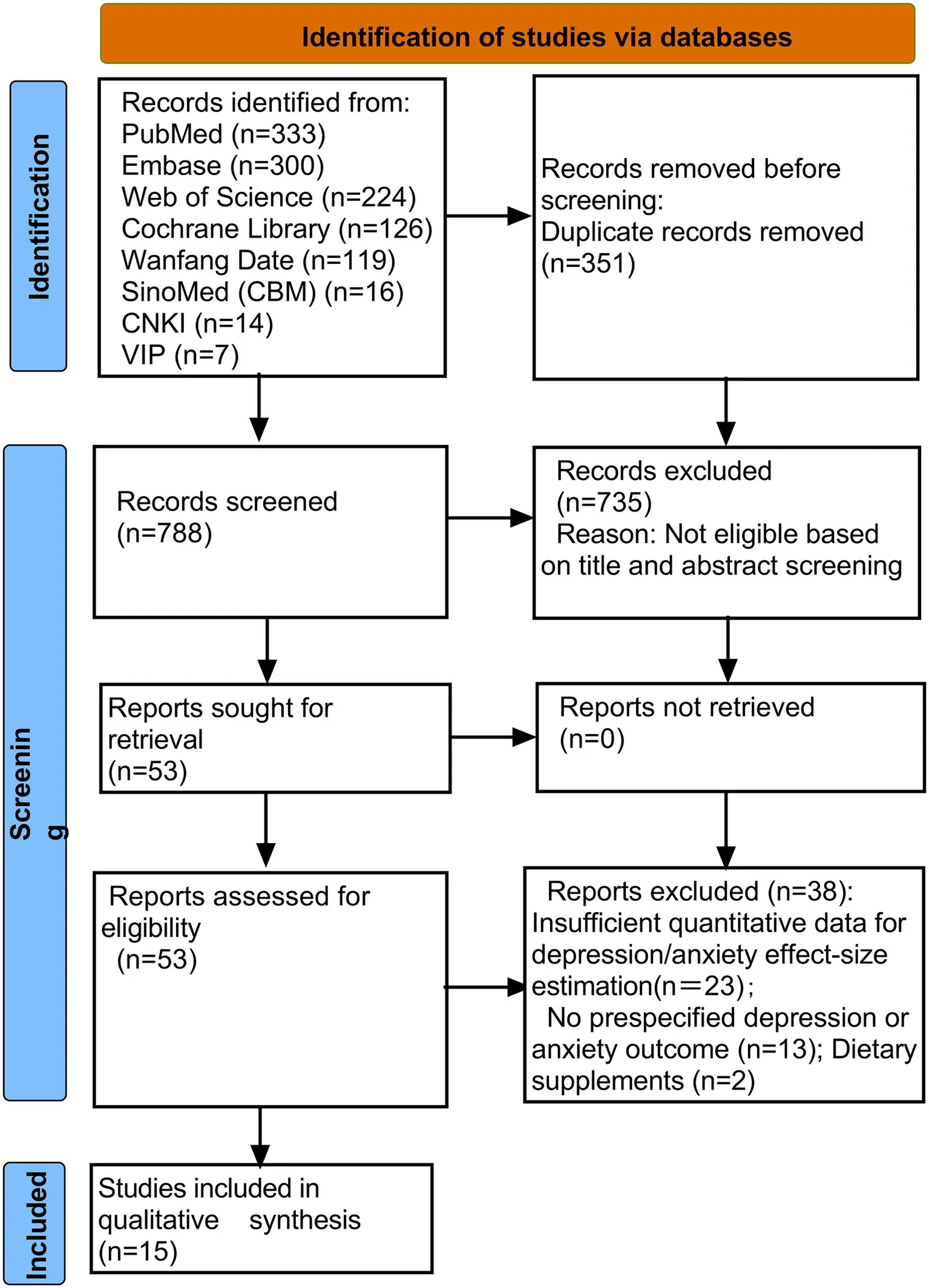
Flow diagram.

Characteristics of the 15 included studies are summarized in Table 1. The studies were published between 1994 and 2025 and were conducted in the United States (*n* = 6) (20–25), China (*n* = 2) (26, 27), Tunisia (*n* = 2) (28, 29), Colombia (*n* = 1) (30), Brazil (*n* = 1) (31), the United Kingdom (*n* = 1) (32), Iran (*n* = 1) (33), and Sweden (*n* = 1) (34). Across the selected quantitative comparisons, 668 unique participants contributed data. Crossover designs predominated, with the remaining studies using parallel-group designs. Study populations included healthy or non-patient adults (21–24, 30, 32, 34), athletic participants (28, 29, 33), healthy school-aged children (25), and participants with psychiatric or other clinical conditions (20, 24, 26, 27, 31, 34). Caffeine was administered as capsules, tablets, coffee, energy drinks, or other caffeinated beverages, with doses ranging from 60 mg/day to 480 mg or expressed relative to body weight. Exposure ranged from acute single-session administration to repeated interventions lasting up to 12 weeks.

**Table 1:** Characteristics of included studies.

Study ID	Country	Participants/sample size	Age (years, T/C; sex)	Duration, design	Caffeine intervention	Control	Washout	Outcomes
Hoppe et al. (34)	Sweden	29 patients with panic disorder	Mean: 24.2 ± 5.1 (overall); 4M/25F	30 min; crossover	Caffeine capsule (150 mg)	Microcrystalline cellulose capsule	2–14 days	Anxiety; NRS anxiety
Hoppe et al. (34)	Sweden	53 healthy controls	Mean: 24.7 ± 6.4 (overall); 20M/33F	30 min; crossover	Caffeine capsule (150 mg)	Microcrystalline cellulose capsule	2–14 days	Anxiety; NRS anxiety
Razazan et al. (33)	Iran	12 elite male wrestlers	Mean: 17.0 ± 1.0 (overall); 12M	60 min; crossover	Caffeine capsule (3 mg/kg BW)	Maltodextrin capsule	2 weeks	Anxiety; STAI
Staack et al. (20)	USA	56 postmenopausal women with overactive bladder	Mean: 69.2 ± 5.0 (overall); 56F	7 days; crossover	Caffeine capsules (200/day)	Identical placebo capsules	NR	Anxiety and depression; BAI, BDI
Yue et al. (26)	China	84 inpatients with depression	T: 37.05 ± 16.57/C: 40.49 ± 15.54	12 weeks; randomized, parallel	Dietary coffee (approx. 95 mg caffeine/day)	Decaffeinated coffee + milnacipran 75 mg/day	NA	Depression; HAMD-24
		T: 43/C: 41						
			T:17M/26F/C: 11M/30F					
Chtourou et al. (28)	Tunisia	19 male physical-education students	Mean: 21.2 ± 1.2 (Overall); 19M	60 min; crossover	Red Bull, 500 mL (160 mg caffeine)	Matched caffeine- and taurine-free beverage	7 days	Anxiety and depression; POMS
Giles et al. (21)	USA	48 healthy adults with low habitual caffeine intake	Mean: 20.12 ± 2.27 (overall); 20M/28F	45 min; crossover	Anhydrous caffeine capsule (400 mg)	Microcrystalline cellulose capsule	≥3 days	Anxiety; STAI
Giles et al. (21)	USA	48 healthy adults with low habitual caffeine intake	Mean: 20.10 ± 4.69 (overall); 20M/28F	45 min; crossover	Anhydrous caffeine capsule (400 mg)	Microcrystalline cellulose capsule	≥3 days	Anxiety; STAI
Liu et al. (27)	China	60 male inpatients with major depression	T: 40.18 ± 7.58/C: 41.15 ± 5.72	4 weeks; randomized, double-blind, parallel	Caffeine tablets (60 mg/day)	Soybean-powder placebo	NA	Depression; HDRS-17
			29M/31F					
		T: 29/C: 31						
Distelberg et al. (22)	USA	49 healthy graduate students	T: 28.1 ± 5.7/C: 26.2 ± 5.8;	5 days; parallel	Regular coffee, 710 mL/day (450 mg/day)	Decaffeinated coffee, 710 mL/day	NA	Anxiety and depression; BAI
		T: 28/C: 21						
								DHP
			T: 11M/17F/C: 7M/14F					
García et al. (30)	Colombia	80 medical students	T: 21.37 ± 2.46/C: 21.7 ± 3.40	60 min; parallel	Energy drinks, 460 mL (147.2–155 mg caffeine)	Carbonated water, 460 mL	NA	Anxiety; STAI
		T: 60/C: 20						
			T: 37M/23F/C: 12M/8F					
Souissi et al. (29)	Tunisia	12 elite judokas	Mean: 21.08 ± 1.16 (overall); sex not report	60 min; crossover	Coffee providing 5 mg/kg BW caffeine	placebo	≥48 h	Anxiety and depression; POMS
Schwartzhaupt et al. (31)	Brazil	19 patients with moderate to severe depression (20 enrolled)	Range: 24–63; 5M/15F	11 h; double-blind crossover	Caffeine pills (450 mg)	Placebo tablets	One recovery night	Depression; HAMD-6
Scott et al. (23)	USA	31 college age men and women	Not report	Pretest; crossover	Caffeine in orange juice (5 mg/kg BW)	Orange juice	Not report	Anxiety; STAI
Quinlan et al. (32)	UK	16 healthy habitual caffeine consumers	M: 36.9 ± 6.3/F: 34.4 ± 11.8; 8M/8F	60 min; randomized crossover	Tea, coffee, or water containing 100 mg caffeine	Corresponding non-caffeinated beverage conditions	1 week	Anxiety; STAI
Tancer et al. (24)	USA	10 panic disorder patients (11 enrolled)	Mean: 32.7 ± 6.6 (overall); 3M/8F	150 min; crossover	Caffeine capsule (480 mg)	Placebo capsule	Not report	Anxiety; STAI
Tancer et al. (24)	USA	11 social Phobia	Mean: 33.3 ± 6.5 (overall); 3M/8F	150 min; crossover	Caffeine capsule (480 mg)	Placebo capsule	Not report	Anxiety; STAI
Tancer et al. (24)	USA	11 healthy volunteers	Mean: 31.6 ± 9.8 (overall); 3M/8F	150 min; crossover	Caffeine capsule (480 mg)	Placebo capsule	Not report	Anxiety; STAI
Bernstein et al. (25)	USA	20 healthy school-age children (21 enrolled)	Mean: 10.6 ± 1.3 (overall); 12M/9F	30 min; crossover	Caffeine in cola (2.5 mg/kg BW)	Caffeine-free cola	1 week	Anxiety; STAI

T, treatment group; C, control group; M, male; F, female; BW, body weight; NA, not available; NRS, 0–100 numerical rating scale; STAI, state–trait anxiety inventory; BAI, Beck anxiety inventory; BDI, Beck depression inventory; HAMD-24, 24-item Hamilton depression scale; POMS, profile of mood states; HDRS-17, 17-item Hamilton rating scale for depression; DHP, Duke health profile; HAMD-6, 6-item Hamilton depression scale.

Three trials assessed depressive symptoms only (26, 27, 31), eight assessed anxiety symptoms only (21, 23–25, 30, 32–34), and four reported both depressive and anxiety outcomes (20, 22, 28, 29). Depressive and anxiety symptoms were assessed using a range of clinician-rated and self-reported instruments. The included studies differed substantially in participant characteristics, caffeine exposure patterns, intervention duration, and outcome assessment. These differences were considered in the subsequent clinically stratified and quantitative syntheses.

### 3.2 Risk of bias

Risk of bias was assessed at the outcome level, with depressive and anxiety outcomes considered separately (Supplementary Figures S1A–D). For depressive outcomes, all seven independent comparisons were judged as having some concerns overall, and none was rated as high risk. The most frequent concerns related to the randomization process, for which six comparisons were rated as having some concerns. Four comparisons raised concerns regarding missing outcome data, and four regarding outcome measurement, while two raised concerns regarding selection of the reported result. Bias due to deviations from intended interventions was judged to be low across all depressive comparisons.

Among the 16 independent anxiety comparisons, three were judged to be at low overall risk of bias and 13 as having some concerns; none was rated as high risk. Concerns were most commonly identified in the randomization process and outcome measurement, affecting 11 and 10 comparisons, respectively. Three comparisons raised concerns regarding missing outcome data, three regarding selection of the reported result, and one regarding deviations from intended interventions. These judgments primarily reflected limited reporting of sequence generation or allocation procedures in several older trials, uncertainty about the maintenance of blinding for subjective anxiety outcomes, and incomplete outcome data or limited prespecification in a small number of comparisons. Detailed domain-level judgments and their supporting rationales are provided in Supplementary Table S4.

### 3.3 Effects of caffeine on depressive symptoms

Seven independent comparisons were available for the broad descriptive synthesis of depressive outcomes. Under a random-effects model using restricted maximum likelihood estimation with Hartung–Knapp adjustment, the overall estimate did not provide clear evidence of a difference in depressive symptom scores between caffeine and control conditions [SMD = −0.49, 95% CI: (−1.33, 0.34), *p* = 0.20]. Considerable between-study variability remained (*I*^2^ = 92.9%, *τ*^2^ = 0.74) (Figure 2), and the corresponding prediction interval was wide [95% PI: (−2.87, 1.88)]. The breadth of this interval was consistent with the marked clinical diversity across trials, particularly with respect to study population, exposure pattern, and the nature of the depressive outcome assessed. The overall estimate was therefore retained as a broad descriptive summary rather than interpreted as a common effect across clinically homogeneous studies.

**Figure 2:**
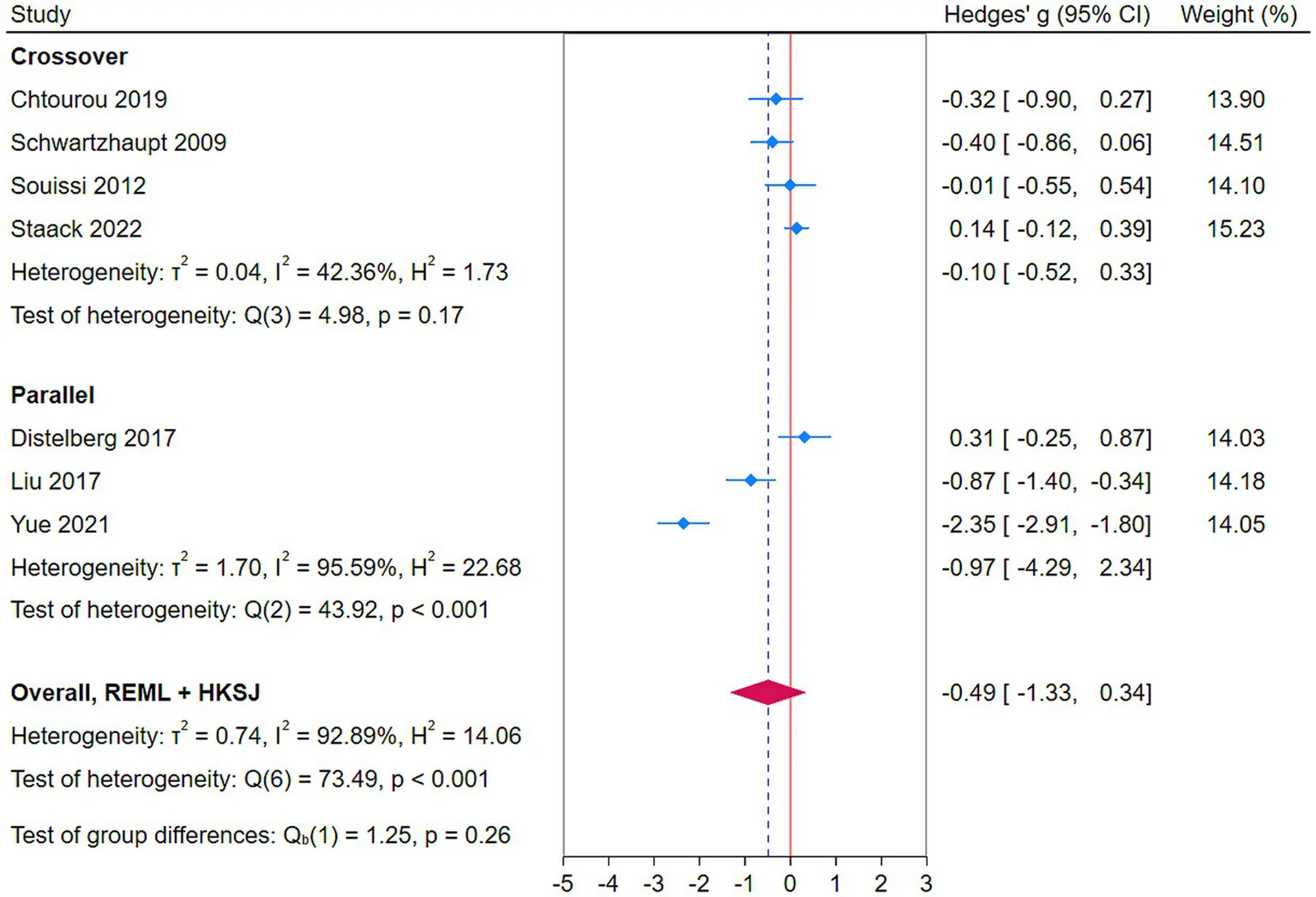
Broad descriptive synthesis of depressive symptoms.

A more clinically coherent subset comprised two acute crossover trials conducted in non-psychiatric participants (28, 29), both of which assessed short-term depression/dejection responses with the Profile of Mood States following single-session caffeine exposure. Quantitative synthesis of these two comparisons did not indicate a clear difference between caffeine and control conditions [SMD = −0.15, 95% CI: (−2.12, 1.81), *p* = 0.51]. Statistical heterogeneity was not detected (*I*^2^ = 0%, *τ*^2^ = 0.00, *p* = 0.45), although the small number of studies and the wide Hartung–Knapp confidence interval substantially limited the precision of the pooled estimate (Supplementary Figure S2).

Evidence from psychiatric populations was more limited and was not combined with the acute non-psychiatric studies. Schwartzhaupt et al. (31) examined an acute caffeine challenge in patients with depression using the clinician-rated HAMD-6. The study-level estimate was compatible with lower depressive symptom scores after caffeine exposure, but remained imprecise and included the null [SMD = −0.40, 95% CI: (−0.86, 0.06)]. Because this comparison differed from the non-psychiatric acute studies in both population and outcome construct, it was considered separately.

For sustained exposure in psychiatric populations, Liu et al. (27) reported lower depressive symptom scores after 4 weeks of caffeine intake [SMD = −0.87, 95% CI: (−1.40, −0.34)], whereas Yue et al. (26) reported a larger estimate after 12 weeks [SMD = −2.35, 95% CI: (−2.91, −1.80)]. Although the direction of effect was similar, the two trials differed appreciably in treatment context, duration of exposure, and magnitude of the observed effect. With only two studies available and limited clinical comparability, a pooled estimate was not considered sufficiently informative, and these findings were summarized narratively.

Repeated caffeine exposure was also examined in two non-psychiatric populations. Staack et al. (20) reported a small positive estimate [SMD = 0.14, 95% CI: (−0.12, 0.39)], and Distelberg et al. (22) reported a similarly small estimate [SMD = 0.31, 95% CI: (−0.25, 0.87)]; neither confidence interval excluded the null. Differences in participant characteristics, intervention protocols, and depressive outcome measures further limited the interpretability of a common pooled effect, and the two studies were therefore described individually.

### 3.4 Effects of caffeine on anxiety symptoms

Sixteen independent comparisons from 12 randomized controlled trials were included in the broad descriptive synthesis of anxiety outcomes. In the random-effects analysis using restricted maximum likelihood estimation with Hartung–Knapp adjustment, anxiety scores were, on average, higher following caffeine exposure than under control conditions [SMD = 0.51, 95% CI: (0.12, 0.89), *p* = 0.01]. Considerable between-study heterogeneity was present (*I*^2^ = 90.3%, *τ*^2^ = 0.43), and the 95% prediction interval extended across the null [95% PI: (−0.95, 1.96)] (Figure 3). The dispersion of effects was consistent with the clinical diversity of the evidence base, particularly with respect to participant characteristics, exposure patterns, and approaches to anxiety assessment. Given this variability, the pooled estimate was interpreted descriptively and not as evidence of a uniform anxiety effect across study settings.

**Figure 3:**
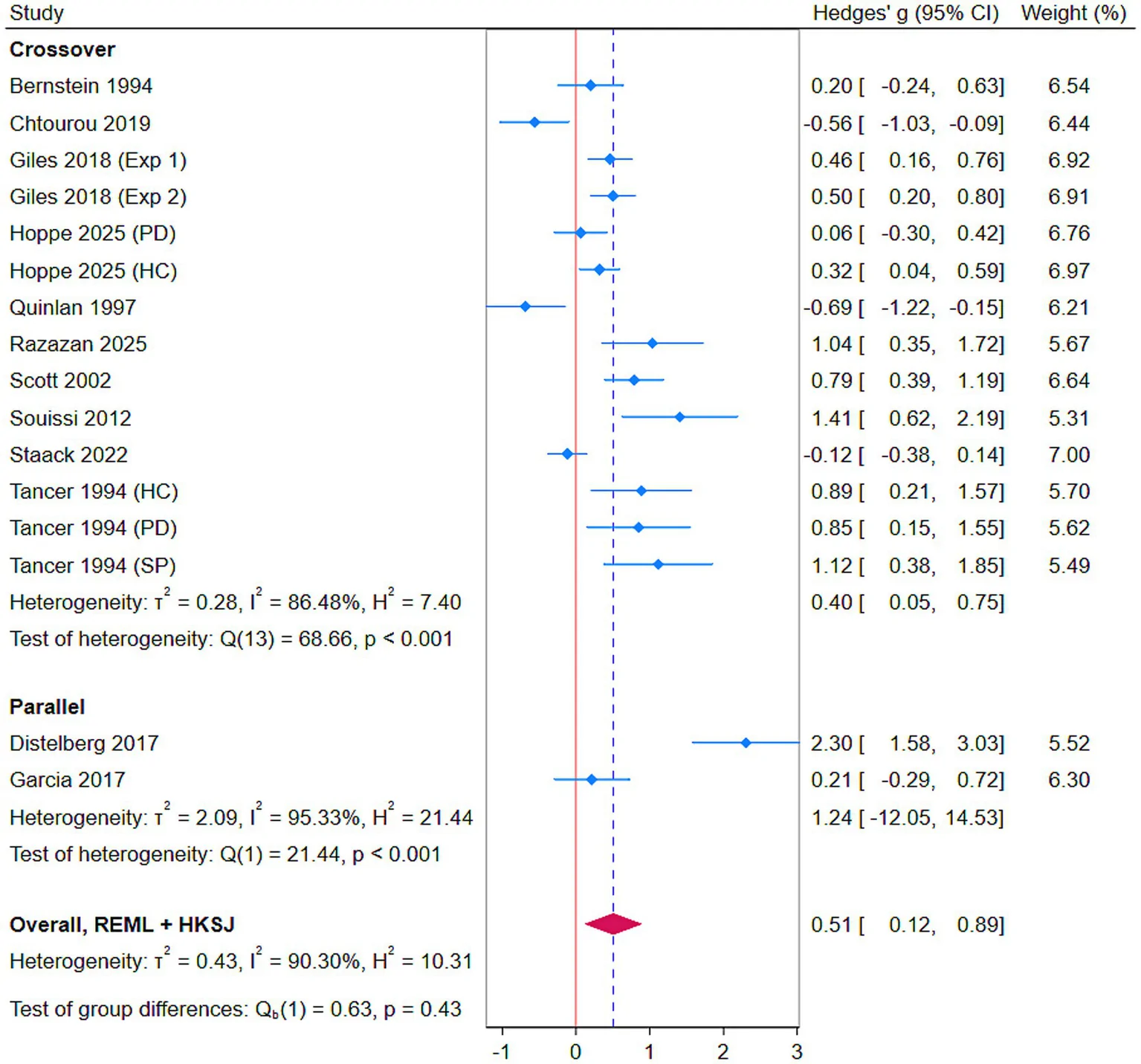
Broad descriptive synthesis of anxiety symptoms.

When the analysis was restricted to 11 acute comparisons from non-psychiatric cohorts reported across 10 publications (21, 23–25, 28–30, 32–34), with Giles et al. (21) contributing two independent experiments and only the healthy-control cohorts from Tancer et al. (24) and Hoppe et al. (34) included in this stratum, the selected outcomes, although assessed using different instruments, predominantly reflected contemporaneous anxiety or anxiety-related mood responses within the acute experimental setting rather than longer-term clinical symptom burden or incident anxiety disorders. These comparisons were therefore considered sufficiently aligned in assessment context to support a descriptive stratified quantitative synthesis. The point estimate remained in the direction of higher anxiety scores, although the confidence interval included the null [SMD = 0.38, 95% CI: (−0.02, 0.79), *p* = 0.06]. Substantial heterogeneity persisted within this more clinically comparable group (*I*^2^ = 85.9%, *τ*^2^ = 0.28, *p* < 0.001), and the prediction interval remained wide [95% PI: (−0.88, 1.65)] (Supplementary Figure S3). These results indicate that restricting the synthesis to acute responses in non-psychiatric participants reduced some sources of clinical variation but did not eliminate the marked variability in the observed anxiety response.

Three additional acute comparisons were available from psychiatric cohorts: two independent cohorts from Tancer et al. (24), comprising patients with panic disorder and social phobia, and the panic-disorder cohort from Hoppe et al. (34). These cohorts were distinct from the healthy-control cohorts from the same publications included in the non-psychiatric synthesis. Because the psychiatric comparisons involved different diagnoses and anxiety measures, quantitative pooling was not considered sufficiently clinically interpretable. The findings were therefore summarized narratively at the study level. Individual effect estimates varied in magnitude across comparisons, and the limited evidence, together with differences in psychiatric populations and outcome constructs, precluded estimation of a common acute anxiety effect of caffeine in psychiatric populations. Individual effect estimates are shown in Figure 3.

Evidence on sustained anxiety outcomes was confined to two studies in non-psychiatric populations and was therefore considered separately. Staack et al. (20) reported an effect estimate close to the null after repeated caffeine exposure [SMD = −0.12, 95% CI: (−0.38, 0.14)], whereas Distelberg et al. (22) reported a substantially larger estimate in the opposite direction [SMD = 2.30, 95% CI: (1.58, 3.03)]. The studies differed not only in the magnitude of the observed effects, but also in participant characteristics and caffeine exposure protocols. In view of these differences and the limited number of available studies, combining the two estimates was unlikely to provide a clinically interpretable summary; the findings were therefore described narratively.

### 3.5 Exploratory subgroup analyses

Two exploratory subgroup analyses were conducted to examine whether variation in effect estimates was associated with caffeine dose or outcome assessment modality. Differences related to exposure pattern and psychiatric status had already been addressed within the outcome-specific clinical syntheses and were therefore not repeated here as conventional subgroup analyses. Dose-stratified analyses were undertaken for both depressive and anxiety outcomes, whereas the analysis by assessment modality was confined to depressive outcomes because the anxiety studies did not provide a meaningful contrast in measurement approach. As several strata contained only a small number of studies and appreciable residual heterogeneity remained in some analyses, the findings were interpreted cautiously and treated as exploratory rather than as evidence of established effect modification.

#### 3.5.1 Stratification by caffeine dose

Caffeine exposure was stratified into exploratory categories of ≤200 mg and >200 mg. The 200-mg boundary was used solely as an analytical grouping criterion to facilitate comparison across trials and was not regarded as an established clinical or biological threshold. For weight-adjusted interventions in which the corresponding absolute dose could not be determined unequivocally at the individual level, classification in the primary analysis was based on the available study-level dose information. These comparisons were subsequently excluded in sensitivity analyses to assess the influence of classification uncertainty. Comparison-level dose assignments and classification certainty are detailed in Supplementary Table S1.

For depressive outcomes, there was no clear evidence of a difference between the two dose categories (test for subgroup differences, *p* = 0.18). The pooled estimate in the ≤ 200 mg category was imprecise and accompanied by substantial heterogeneity [SMD = −0.84, 95% CI: (−2.56, 0.89), *p* = 0.22; *I*^2^ = 95.3%, *τ*^2^ = 1.11], whereas the estimate in the >200 mg category was close to the null [SMD = −0.06, 95% CI: (−0.95, 0.83), *p* = 0.81; *I*^2^ = 47.3%, *τ*^2^ = 0.06] (Supplementary Figure S4). Souissi et al. (29) reported caffeine exposure in mg/kg and could not be assigned unequivocally to either side of the 200-mg boundary at the individual level. Exclusion of this comparison produced little change in the between-category comparison, which remained non-significant (*p* = 0.23).

For anxiety outcomes, the pooled estimates differed between the two exploratory dose categories (test for subgroup differences, *p* < 0.001). The ≤ 200 mg category yielded an estimate close to the null [SMD = −0.05, 95% CI: (−0.41, 0.30), *p* = 0.72; *I*^2^ = 71.8%, *τ*^2^ = 0.09], whereas the >200 mg category yielded a higher pooled anxiety estimate [SMD = 0.97, 95% CI: (0.55, 1.39), *p* < 0.001; *I*^2^ = 76.2%, *τ*^2^ = 0.20] (Figure 4). Four comparisons (23, 25, 29, 33) used weight-adjusted caffeine doses that could not be classified unequivocally relative to the 200-mg boundary at the individual level. Restricting the analysis to comparisons with unambiguous dose classification yielded a similar pattern: the estimate remained close to the null in the ≤200 mg category [SMD = −0.10, 95% CI: (−0.52, 0.32)] and remained positive in the >200 mg category [SMD = 0.97, 95% CI: (0.27, 1.67)]. The unrounded pooled estimate in the > 200 mg category changed slightly from 0.972 in the primary analysis to 0.968 in the restricted sensitivity analysis, although both values round to 0.97 when reported to two decimal places. The between-category difference also remained statistically significant (*p* = 0.001). The persistence of this contrast after exclusion of uncertain classifications suggests that the observed difference was not attributable solely to the handling of weight-adjusted doses. Nevertheless, substantial residual heterogeneity remained within both dose categories, and the analysis relied on categorical rather than continuous dose information. The results should therefore be interpreted as exploratory and do not establish a continuous dose–response relationship or support 200 mg as a clinical threshold.

**Figure 4:**
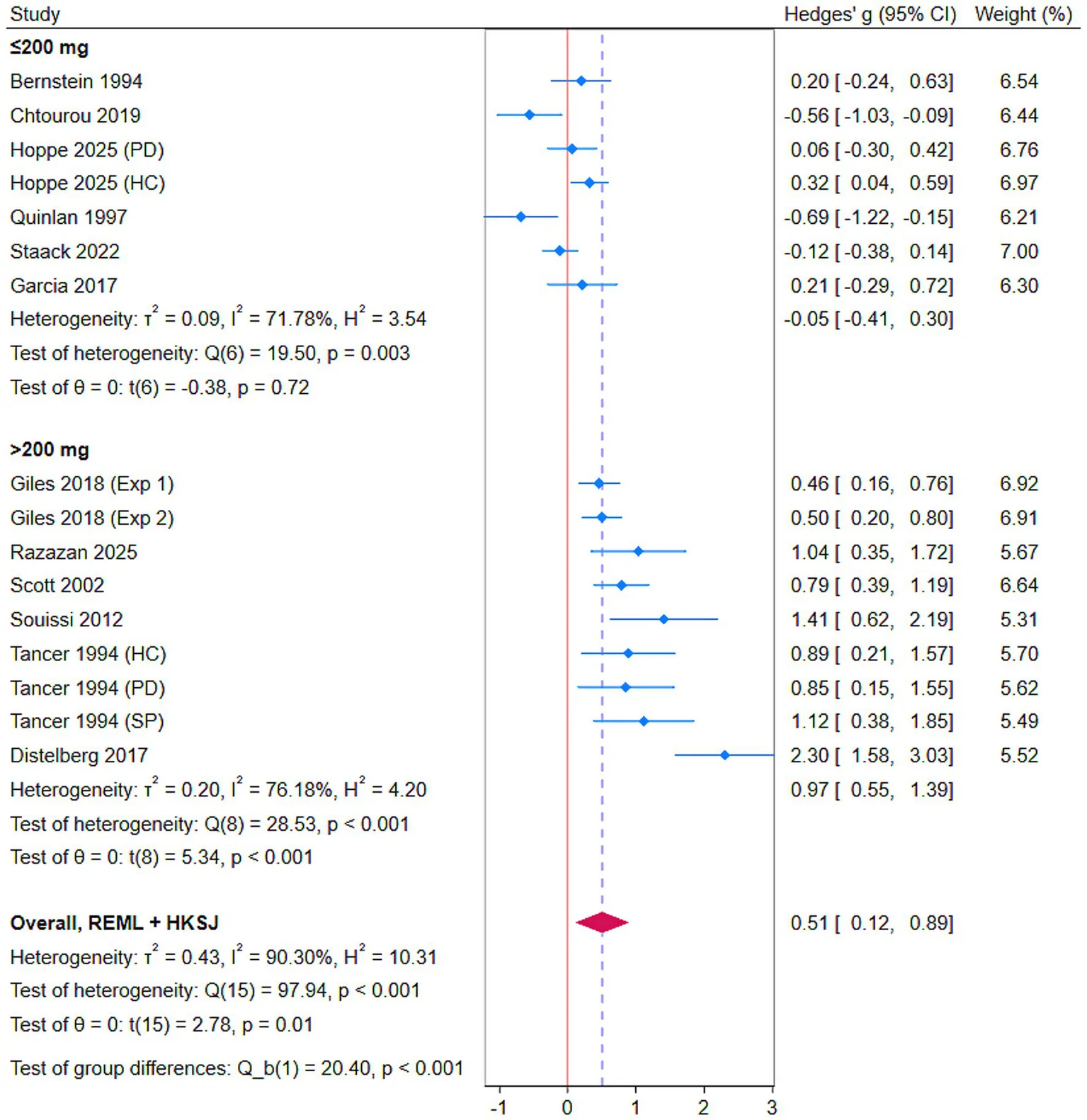
Exploratory dose-stratified analysis of anxiety symptoms. This forest plot presents the exploratory comparison of effect estimates across the two dose categories and the residual heterogeneity within each category. The findings should not be interpreted as evidence of a continuous dose–response relationship or a clinical threshold at 200 mg.

#### 3.5.2 Exploratory analysis by assessment modality

For depressive outcomes, effect estimates were explored according to assessment modality. Studies using self-reported measures yielded an estimate close to the null [SMD = 0.08, 95% CI: (−0.23, 0.40), *p* = 0.45], with little evidence of between-study heterogeneity (*I*^2^ = 0%, *τ*^2^ = 0.00). In contrast, studies using clinician-rated measures produced a more negative, but markedly less precise, pooled estimate [SMD = −1.20, 95% CI: (−3.73, 1.33), *p* = 0.18], together with substantial heterogeneity (*I*^2^ = 93.4%, *τ*^2^ = 0.97) (Supplementary Figure S5). The subgroup contrast reached statistical significance (*p* = 0.03); however, assessment modality was closely aligned with other study characteristics. Clinician-rated outcomes were drawn predominantly from psychiatric populations and more clinically oriented intervention settings, whereas self-reported measures were more common in non-psychiatric samples. The observed difference therefore cannot be attributed to measurement modality alone and is best regarded as an exploratory finding reflecting several overlapping clinical and methodological features. A corresponding analysis was not undertaken for anxiety outcomes because the included comparisons relied on participant-reported anxiety measures, leaving no informative contrast by assessment modality.

### 3.6 Sensitivity analysis

#### 3.6.1 Analysis according to study design

Study design was examined as a methodological source of variation in the pooled estimates. For depressive outcomes, crossover trials yielded an effect estimate close to the null [SMD = −0.10, 95% CI: (−0.52, 0.33)], with moderate heterogeneity (*I*^2^ = 42.4%, *τ*^2^ = 0.04). The estimate from parallel-group trials was more negative but markedly less precise [SMD = −0.97, 95% CI: (−4.29, 2.34)], and substantial heterogeneity remained (*I*^2^ = 95.6%, *τ*^2^ = 1.70). The test for subgroup differences did not indicate a clear difference between crossover and parallel-group designs (*p* = 0.26).

For anxiety outcomes, the pooled estimate from crossover trials was positive [SMD = 0.40, 95% CI: (0.05, 0.75)], although considerable heterogeneity persisted (*I*^2^ = 86.5%, *τ*^2^ = 0.28). Parallel-group trials produced a larger point estimate, but with considerable uncertainty [SMD = 1.24, 95% CI: (−12.05, 14.53)], together with substantial heterogeneity (*I*^2^ = 95.3%, *τ*^2^ = 2.09). No clear between-design difference was detected (*p* = 0.43). The exceptionally wide confidence interval for the parallel-group estimate reflects the very small number of contributing comparisons and limits the extent to which differences between study designs can be interpreted.

The available data therefore provide little support for study design as an independent explanation for the variation in depressive or anxiety effect estimates. Given the sparse evidence within the parallel-group strata and the residual heterogeneity within both designs, these analyses were retained as methodological assessments rather than interpreted as evidence of design-specific effects.

#### 3.6.2 Sensitivity analysis using alternative within-participant correlations

For crossover comparisons without reported paired variance information, sensitivity analyses were conducted using alternative within-participant correlations of *r* = 0.25 and *r* = 0.75, with *r* = 0.50 retained for the primary analysis. Reported adjusted within-participant estimates and their corresponding standard errors were used directly where available and were therefore not affected by these assumptions. The pooled estimates for both depressive and anxiety outcomes changed only minimally across the alternative correlation assumptions, with no material change in the direction or statistical interpretation of the results (Supplementary Table S5).

#### 3.6.3 Analysis restricted to isolated caffeine interventions

To examine whether the primary findings were sensitive to the inclusion of caffeine-containing beverages and multi-component formulations, the analyses were repeated after restriction to comparisons in which caffeine was the differential active component between intervention and control conditions. For depressive outcomes, the restricted estimate remained in the same direction as the primary analysis but was attenuated and substantially less precise, with considerable residual heterogeneity. For anxiety outcomes, the restricted estimate remained positive and was similar in magnitude to the primary estimate; between-study heterogeneity was reduced, although appreciable variability remained. Restriction to isolated caffeine contrasts therefore did not materially alter the statistical interpretation of either outcome, while the smaller evidence base, particularly for depression, increased uncertainty in the corresponding estimates (Supplementary Table S6).

#### 3.6.4 Leave-one-out sensitivity analysis

Leave-one-out analyses were performed to assess the extent to which the pooled estimates were influenced by individual independent comparisons. For depressive outcomes, sequential omission of each comparison produced no material change in the overall statistical interpretation. The pooled estimates remained negative throughout, and all corresponding 95% confidence intervals continued to include the null. The greatest attenuation was observed after exclusion of Yue et al. (26), suggesting that this comparison contributed appreciably to the magnitude of the pooled estimate, although its removal did not alter the overall inference. For anxiety outcomes, the pooled estimates remained positive and statistically significant after omission of every individual comparison. The largest attenuation occurred after exclusion of Distelberg et al. (22); nevertheless, the resulting confidence interval remained above the null. Thus, while individual comparisons contributed to variation in the magnitude of the pooled effects, no single comparison appeared to determine the overall statistical interpretation of either depressive or anxiety outcomes (Supplementary Table S7).

### 3.7 Assessment of small-study effects and publication bias

For depressive outcomes, the funnel plot showed some visual asymmetry in the distribution of effect estimates (Supplementary Figure S6). Interpretation was limited, however, by the small evidence base of only seven independent comparisons, for which funnel-plot patterns are particularly susceptible to random variation and individual influential estimates. Formal testing for funnel-plot asymmetry was therefore not considered sufficiently informative, and no inference regarding the presence or absence of publication bias was drawn from the depressive dataset. For anxiety outcomes, the funnel plot also appeared asymmetric (Supplementary Figure S7), and Egger’s regression test indicated evidence of small-study effects (*z* = 2.740, *p* = 0.006). This finding warrants caution in interpreting the pooled anxiety estimate, although the observed asymmetry is not specific to publication bias. Given the substantial clinical and statistical heterogeneity across the anxiety comparisons, differences in participant populations, exposure characteristics, outcome assessment, and study precision may also have contributed to the observed pattern.

### 3.8 Certainty of evidence

For depressive symptoms, the certainty of evidence was rated as very low. The evidence was downgraded for risk of bias, substantial inconsistency, indirectness arising from differences in populations, exposure patterns, and outcome measures, and imprecision of the pooled estimate [SMD = −0.49, 95% CI: (−1.33, 0.34), *p* = 0.20]. Publication bias was not further downgraded for depressive outcomes because only seven independent comparisons were available, precluding a reliable assessment of funnel-plot asymmetry. This decision reflects insufficient evidence to justify additional downgrading rather than evidence that publication bias was absent.

For anxiety symptoms, the certainty of evidence was also rated as very low. The evidence was downgraded for risk of bias, substantial inconsistency, indirectness across populations, exposure patterns, and outcome measures, and imprecision despite the pooled estimate excluding the null [SMD = 0.51, 95% CI: (0.12, 0.89), *p* = 0.01]. Publication bias was additionally downgraded because funnel-plot asymmetry and evidence of small-study effects raised sufficient concern for publication bias, although substantial between-study heterogeneity may also have contributed to the observed asymmetry. Detailed GRADE assessments for depressive and anxiety outcomes are presented in Supplementary Table S8.

## 4 Discussion

### 4.1 Principal findings

The present findings do not support a uniform effect of caffeine on depressive or anxiety symptoms across clinical and experimental settings. For depressive symptoms, the broad pooled estimate did not show a clear and consistent difference between caffeine and control conditions. Anxiety scores were higher on average following caffeine exposure, but the prediction interval crossed the null, indicating substantial variation across study settings. Given the very low certainty of evidence for both outcomes, these pooled estimates are better interpreted as broad summaries of a heterogeneous evidence base than as effects expected to apply uniformly across populations.

For depressive symptoms, the non-significant broad pooled estimate should not be interpreted as evidence of the absence of a clinically relevant effect, particularly given the wide confidence and prediction intervals and the very-low certainty of the evidence. Acute studies in non-psychiatric participants showed little indication of a reproducible short-term change (28, 29), whereas studies in psychiatric populations differed substantially in treatment context and exposure duration (26, 27, 31). In particular, trials conducted alongside antidepressant therapy are not directly comparable with isolated caffeine exposure. Repeated-exposure studies in non-psychiatric populations also produced small and imprecise estimates (20, 22). The main contribution of the present analysis is therefore to show that randomized evidence does not support a consistent antidepressant effect across distinct populations and exposure contexts. This may partly explain the difference from observational evidence linking habitual coffee or caffeine intake with lower depressive symptoms (35).

Interpretation of the anxiety findings likewise depends on study context. Acute non-psychiatric comparisons generally produced smaller and more variable effects than the broad pooled estimate (21, 23–25, 28–30, 32–34), while evidence from psychiatric populations remained limited and imprecise (24, 34). Sustained/repeated-exposure studies also yielded divergent findings (20, 22). Accordingly, although the broad pooled estimate for anxiety was statistically significant, this finding should not be interpreted as evidence of clinically meaningful deterioration or an increased incidence of anxiety disorders, particularly because most included trials assessed continuous symptom scores rather than diagnostic outcomes. Although exploratory dose stratification suggested larger anxiety estimates in the >200 mg category, residual heterogeneity and categorical dose classification do not support interpretation of 200 mg as a clinical threshold or evidence of a continuous dose–response relationship. Overall, the available randomized evidence is more consistent with context-dependent variation in affective response than with a fixed effect of caffeine on depressive or anxiety symptoms.

### 4.2 Pharmacological mechanisms

The heterogeneous affective responses to caffeine may be partly understood in the context of its actions on central adenosine signaling. Caffeine acts primarily as a non-selective antagonist of adenosine A_1_ and A_2A_ receptors, thereby influencing neuronal excitability and neurotransmitter systems involved in arousal and affective regulation (36). Acute antagonism of adenosine signaling may alter dopaminergic and serotonergic neurotransmission, providing a plausible biological basis for short-term changes in mood-related responses. With repeated exposure, compensatory adaptations in adenosine signaling may attenuate or modify these acute effects (37). Such neuroadaptation, together with differences in habitual caffeine use and baseline psychological status, may contribute to the variation observed between acute and sustained-exposure studies rather than implying a uniform antidepressant action.

For anxiety-related responses, adenosine receptor antagonism may increase central arousal and sympathetic activation, which could heighten anxiety symptoms in susceptible individuals. Greater caffeine exposure may intensify these physiological responses, offering a possible explanation for the larger anxiety estimates observed in the higher-dose category. This interpretation remains provisional, however, because the present analysis used categorical dose groupings and did not establish a continuous dose–response relationship or a biological threshold. Interindividual differences in caffeine sensitivity, metabolism, habitual intake, and baseline anxiety vulnerability may further modify these responses. The available mechanistic evidence therefore provides a biologically plausible framework for the heterogeneity observed across trials, but does not by itself establish that the differences between dose categories are causal.

### 4.3 Strengths and limitations

Several methodological features strengthen the rigor of the present review. Restricting eligibility to randomized controlled trials reduced the influence of lifestyle and behavioral confounding that complicates the interpretation of observational evidence. Clinical heterogeneity was addressed by distinguishing acute from sustained exposure and psychiatric from non-psychiatric populations where the evidence allowed, while clinically dissimilar studies were summarized narratively rather than pooled solely on the basis of statistical standardization. Explicit selection and handling procedures were applied to multi-arm, crossover, and repeated-measure designs to minimize double-counting and inappropriate weighting. The analytical approach incorporated REML estimation, Hartung–Knapp adjustment, prediction intervals, and sensitivity analyses examining key assumptions and influential comparisons. Risk of bias was assessed at the outcome level, and certainty of evidence was evaluated using GRADE.

The principal limitation is the size and diversity of the available evidence base. Fifteen trials, comprising 668 unique participants contributing to the selected quantitative comparisons, were included, and several clinically relevant strata contained only a small number of studies. Considerable heterogeneity persisted even within more clinically comparable subsets. Differences in psychiatric status, participant characteristics, caffeine formulation, exposure duration, habitual caffeine use, and outcome assessment may all have contributed to this variability. Potential biological modifiers were also poorly characterized. Genetic variation in adenosine-related pathways has been associated with caffeine-induced anxiety in experimental studies (38, 39), but such factors were seldom reported in the included trials and could not be examined as potential sources of heterogeneity. In addition, the use of different psychometric instruments raises a construct-level issue that cannot be resolved by standardization alone: measures of transient mood states and clinical symptom severity are not necessarily interchangeable. These features limit both the precision of the pooled estimates and their generalizability across clinical settings.

Methodological limitations within the primary studies add further uncertainty. All 7 depressive comparisons were judged to have some concerns for risk of bias; among the 16 anxiety comparisons, 13 had some concerns and three were judged to be at low risk. The main concerns involved incomplete reporting of randomization procedures, outcome measurement, missing data, and selective reporting, particularly in older trials. Several studies used crossover designs, and paired variance information was unavailable for some comparisons. Although alternative within-participant correlation assumptions produced little change in the overall interpretation, uncertainty related to carry-over effects, washout adequacy, and unreported covariance cannot be excluded. The inclusion of caffeine-containing beverages and multi-component formulations also introduced some exposure heterogeneity, although analyses restricted to contrasts in which caffeine was the differential active component did not materially change the overall interpretation.

These limitations are reflected in the certainty assessments. Evidence for both depressive and anxiety outcomes was rated as very low because of concerns regarding risk of bias, inconsistency, indirectness, and imprecision; for anxiety, funnel-plot asymmetry and evidence of small-study effects also raised concern about publication bias. Most trials evaluated relatively short-term exposure, which limits inference regarding sustained habitual caffeine consumption, and randomized evidence in children and adolescents was sparse. The findings should therefore be interpreted within these evidential boundaries rather than as estimates of uniform or long-term clinical effects.

### 4.4 Implications for clinical practice and future research

The present findings do not provide a basis for defining a clinically harmful caffeine dose or for modifying existing population-level intake recommendations. Given the substantial heterogeneity, prediction intervals spanning the null, and very-low-certainty evidence, the pooled estimates should not be translated into uniform clinical guidance. The observed increase in anxiety scores should not be taken to indicate clinically meaningful deterioration or an increased incidence of anxiety disorders, while the non-significant depressive estimate does not exclude a clinically relevant effect. Because much of the randomized evidence was derived from acute or short-term experimental caffeine exposure, these findings should not be extrapolated directly to habitual dietary caffeine consumption or long-term psychiatric outcomes. In clinical practice, caffeine-related symptoms may therefore be interpreted cautiously in relation to habitual intake, individual sensitivity, and their temporal relationship with caffeine exposure rather than on the basis of a universal dose threshold.

Future research should place greater emphasis on clinically coherent study populations, clearly defined exposure patterns, and outcomes with established clinical relevance. Longer-duration randomized trials, where feasible, are needed to determine whether short-term affective responses persist with sustained caffeine exposure. Greater consistency in the reporting of habitual caffeine intake, withdrawal status, caffeine formulation, dose, and timing would also improve comparability across studies. Studies incorporating clinically interpretable outcome thresholds, rather than symptom scores alone, would help clarify whether observed changes translate into meaningful effects for patients. Biological sources of interindividual variability, including caffeine metabolism and adenosine-related pathways such as *CYP1A2* and *ADORA2A*, may also warrant investigation, but such analyses should be considered complementary to, rather than substitutes for, well-designed clinical trials.

## 5 Conclusion

This systematic review and meta-analysis of randomized controlled trials found no clear evidence of a consistent effect of caffeine on depressive symptoms across populations and exposure settings, although the non-significant pooled estimate does not exclude a clinically relevant effect. Anxiety scores were higher on average following caffeine exposure, but substantial heterogeneity, a prediction interval spanning the null, and very-low-certainty evidence limit the clinical interpretation of this finding and do not establish clinically meaningful deterioration or an increased incidence of anxiety disorders. Exploratory dose-stratified findings do not support a clinical threshold or continuous dose–response relationship. Because much of the evidence was derived from acute or short-term experimental exposure, the findings should not be extrapolated directly to habitual caffeine consumption or long-term psychiatric outcomes. Further well-designed trials with clinically meaningful outcomes and longer follow-up are warranted.

## Data Availability

The data extracted and analyzed during this study are provided in the article and its Supplementary material. The complete study- and comparison-level extraction dataset, including participant counts, numerical data used for effect-size estimation, and the corresponding effect-size estimates, is provided in Supplementary Table S2. Supplementary Table S1 documents dose-classification decisions, while Supplementary Table S3 provides the audit trail for the selection of analytical comparisons and the handling of shared controls and repeated measurements. Supplementary Table S4 presents the risk-of-bias assessments, Supplementary Tables S5–S7 report the sensitivity analyses, and Supplementary Table S8 presents the GRADE certainty-of-evidence assessment. Further inquiries regarding the data may be directed to the corresponding authors.
