# Supplementary material for: Effects of caffeine intake on depressive and anxiety symptoms: a systematic review and meta-analysis of randomized controlled trials
Source: Front Nutr. 2026 Oct 29;13:1950050. doi: 10.3389/fnut.2026.1950050 (PMC13755324; doi:10.3389/fnut.2026.1950050)
Supplement: Supplementary file 1 [file Data_Sheet_1.docx]

Supplementary Material

# 1 Supplementary Figures and Tables

## 1.1 Supplementary Table S1

**Supplementary Table S1. Comparison-level caffeine-dose classification for exploratory dose-stratified analyses**

| **Study / comparison** | **Outcome(s)** | **Reported caffeine dose** | **Selected analytical dose / handling of multiple doses** | **Study-level information used for classification** | **Primary exploratory category** | **Classification certainty** | **Classification rationale** | **Restricted sensitivity analysis** |
| --- | --- | --- | --- | --- | --- | --- | --- | --- |
| Hoppe, 2025 (PD cohort) | Anxiety | 150 mg | 150 mg caffeine vs placebo; PD cohort analyzed separately from HC cohort. | Absolute dose directly reported. | ≤200 mg | Unambiguous | Selected dose is explicitly 150 mg and lies below the exploratory 200-mg boundary. | Included |
| Hoppe, 2025 (HC cohort) | Anxiety | 150 mg | 150 mg caffeine vs placebo; HC cohort analyzed separately from PD cohort. | Absolute dose directly reported. | ≤200 mg | Unambiguous | Selected dose is explicitly 150 mg and lies below the exploratory 200-mg boundary. | Included |
| Razazan, 2025 | Anxiety | 3 mg/kg | Caffeine-alone condition vs placebo; L-theanine and caffeine + L-theanine conditions excluded. | Mean body mass 70.9 ± 11.8 kg; study-level mean equivalent ≈212.7 mg (3 mg/kg × 70.9 kg). | >200 mg* | Uncertain | The study-level mean equivalent is above 200 mg, but participant-level absolute doses cannot be determined unequivocally and may cross the boundary. | Excluded |
| Staack, 2022 | Anxiety; depression | 200 or 400 mg/day | 200 mg/day vs placebo retained; 400 mg/day not entered independently because the same crossover participants and placebo were reused. | Absolute doses directly reported. | ≤200 mg | Unambiguous | The selected analytical dose is exactly 200 mg/day and was assigned to the ≤200-mg category. | Included |
| Yue, 2021 | Depression | ≈95 mg/day | Caffeinated coffee + milnacipran 75 mg/day vs matched decaffeinated coffee + milnacipran 75 mg/day at Week 12. | Approximate absolute caffeine dose directly reported for the selected intervention. | ≤200 mg | Unambiguous | The selected caffeine exposure is approximately 95 mg/day, below the exploratory boundary. | Included |
| Chtourou, 2019 | Anxiety; depression | 160 mg | Red Bull vs matched caffeine- and taurine-free beverage at 60 min. | Absolute caffeine dose directly reported. | ≤200 mg | Unambiguous | The selected intervention contains 160 mg caffeine, below the exploratory boundary. | Included |
| Giles, 2018 (Experiment 1) | Anxiety | 400 mg | Caffeine vs placebo at 45 min; 75-min assessment not entered independently. | Absolute dose directly reported. | >200 mg | Unambiguous | The selected dose is explicitly 400 mg, above the exploratory boundary. | Included |
| Giles, 2018 (Experiment 2) | Anxiety | 400 mg | Caffeine vs placebo at 45 min; analyzed as a genuinely independent experiment. | Absolute dose directly reported. | >200 mg | Unambiguous | The selected dose is explicitly 400 mg, above the exploratory boundary. | Included |
| Liu, 2017 | Depression | 60 or 120 mg/day | 60 mg/day + escitalopram vs placebo + escitalopram retained at Week 4; 120-mg arm not entered independently. | Absolute doses directly reported. | ≤200 mg | Unambiguous | The selected analytical dose is 60 mg/day, below the exploratory boundary. | Included |
| Distelberg, 2017 | Anxiety; depression | ≈450 mg/day (control ≈12 mg/day) | Regular caffeinated coffee vs decaffeinated coffee at Treatment Day 5. | Approximate absolute daily caffeine exposure directly reported. | >200 mg | Unambiguous | The selected intervention exposure is approximately 450 mg/day, above the exploratory boundary; the control contained only residual caffeine (~12 mg/day). | Included |
| García, 2017 | Anxiety | A: 149.5 mg; B: 147.2 mg; C: 155 mg | Energy-drink arms A+B+C combined before comparison with the shared carbonated-water control. | Absolute caffeine content directly reported for all three eligible intervention arms. | ≤200 mg | Unambiguous | All three combined intervention arms lie below 200 mg; combining the arms therefore does not create dose-category ambiguity. | Included |
| Souissi, 2012 | Anxiety; depression | 5 mg/kg | Caffeinated coffee vs placebo at 60 min. | Mean body mass 83.75 ± 20.2 kg; study-level mean equivalent ≈418.8 mg (5 mg/kg × 83.75 kg). | >200 mg* | Uncertain | The study-level mean equivalent is well above 200 mg, but individual absolute doses were not reported and therefore cannot be classified unequivocally. | Excluded |
| Schwartzhaupt, 2009 | Depression | 450 mg | Caffeine tablets vs placebo during total sleep deprivation; morning endpoint retained. | Absolute dose directly reported. | >200 mg | Unambiguous | The selected dose is explicitly 450 mg, above the exploratory boundary. | Included |
| Scott, 2002 | Anxiety | 5 mg/kg | Caffeine in orange juice vs orange-juice placebo; pretest anxiety assessment retained. | 5 mg/kg in 31 college-age men and women; participant-level and summary body-weight data were not reported. | >200 mg* | Uncertain | The comparison was assigned to the >200-mg category in the primary exploratory analysis on the basis of the adult study population and the 5-mg/kg regimen; however, because body-weight data were not reported, the absolute dose relative to the 200-mg boundary could not be verified unequivocally. | Excluded |
| Quinlan, 1997 | Anxiety | 100 mg | Study-defined ±caffeine contrast across beverage conditions at 60 min. | Absolute caffeine dose directly reported. | ≤200 mg | Unambiguous | The caffeinated conditions contained 100 mg caffeine, below the exploratory boundary. | Included |
| Tancer, 1994 (HC cohort) | Anxiety | 480 mg | Caffeine vs placebo at 90 min; HC cohort analyzed independently. | Absolute dose directly reported. | >200 mg | Unambiguous | The selected dose is explicitly 480 mg, above the exploratory boundary. | Included |
| Tancer, 1994 (PD cohort) | Anxiety | 480 mg | Caffeine vs placebo at 90 min; PD cohort analyzed independently. | Absolute dose directly reported. | >200 mg | Unambiguous | The selected dose is explicitly 480 mg, above the exploratory boundary. | Included |
| Tancer, 1994 (SP cohort) | Anxiety | 480 mg | Caffeine vs placebo at 90 min; social-phobia cohort analyzed independently. | Absolute dose directly reported. | >200 mg | Unambiguous | The selected dose is explicitly 480 mg, above the exploratory boundary. | Included |
| Bernstein, 1994 | Anxiety | 2.5 or 5.0 mg/kg | 2.5 mg/kg vs placebo retained; 5.0-mg/kg condition not entered independently. | Mean body weight 38.1 ± 12.5 kg; study-level mean equivalent ≈95.3 mg (2.5 mg/kg × 38.1 kg). | ≤200 mg* | Uncertain | The study-level mean equivalent is below 200 mg, but participant-level absolute doses were not reported; unequivocal classification of every participant is therefore not possible. | Excluded |

**Note.** The 200-mg boundary was used solely as an exploratory analytical grouping criterion and should not be interpreted as an established clinical or biological threshold. ***Unambiguous*** indicates that the selected analytical caffeine dose was reported or could be classified unequivocally as falling on one side of the 200-mg boundary. ***Uncertain*** indicates a weight-adjusted regimen for which participant-level absolute caffeine doses could not be established unequivocally from the report. ***For multi-dose or multi-arm trials***, only the comparison selected according to the study-level rules in Supplementary Table S3 was classified; additional correlated dose contrasts were not entered independently. Comparisons with uncertain classification were retained in the primary exploratory dose-stratified analysis using the available study-level information but were excluded from the sensitivity analysis restricted to unambiguous classifications. ***** Primary exploratory category assigned using available study-level information despite uncertain classification.

**Abbreviations:** PD, panic disorder; HC, healthy control; SP, social phobia.

## 1.2 Supplementary Table S2

**Supplementary Table S2. Numerical extraction and study-level effect-size calculations**

| **Study / cohort** | **Outcome** | **Caffeine N** | **Caffeine mean (SD)** | **Control N** | **Control mean (SD)** | **Effect-size / variance handling** | **Hedges' g** | **SE** | **95% CI** |
| --- | --- | --- | --- | --- | --- | --- | --- | --- | --- |
| Chtourou 2019 | Depression | 19 | 4.80 (2.21) | 19 | 6.10 (5.10) | Paired variance unavailable; primary SE estimated using *r*=0.50 | -0.317 | 0.299 | [-0.90, 0.27] |
| Schwartzhaupt 2009 | Depression | 19 | 4.40 (3.40) | 19 | 5.60 (2.40) | Paired variance unavailable; primary SE estimated using *r*=0.50 | -0.399 | 0.234 | [-0.86, 0.06] |
| Souissi 2012 | Depression | 12 | 0.55 (1.66) | 12 | 0.56 (1.04) | Paired variance unavailable; primary SE estimated using *r*=0.50 | -0.007 | 0.279 | [-0.55, 0.54] |
| staack 2022 | Depression | 56 | 7.51 (5.69) | 56 | 7.66 (5.69) | Reported within-participant adjusted estimate and SE used directly | 0.135 | 0.132 | [-0.12, 0.39] |
| Distelberg 2017 | Depression | 28 | 28.30 (17.80) | 21 | 23.50 (11.00) | Independent-group variance | 0.309 | 0.286 | [-0.25, 0.87] |
| Liu 2017 | Depression | 29 | 8.44 (1.43) | 31 | 9.71 (1.45) | Independent-group variance | -0.870 | 0.270 | [-1.40, -0.34] |
| Yue 2021 | Depression | 43 | 9.14 (3.99) | 41 | 18.80 (4.14) | Independent-group variance | -2.355 | 0.284 | [-2.91, -1.80] |
| Bernstein 1994 | Anxiety | 20 | 28.80 (4.10) | 20 | 28.00 (3.80) | Paired variance unavailable; primary SE estimated using *r*=0.50 | 0.198 | 0.222 | [-0.24, 0.63] |
| Chtourou 2019 | Anxiety | 19 | 8.30 (2.90) | 19 | 10.80 (5.30) | Paired variance unavailable; primary SE estimated using *r*=0.50 | -0.560 | 0.238 | [-1.03, -0.09] |
| Giles 2018 (Exp 1) | Anxiety | 48 | 42.98 (9.91) | 48 | 38.69 (8.52) | Paired variance unavailable; primary SE estimated using *r*=0.50 | 0.461 | 0.151 | [0.16, 0.76] |
| Giles 2018 (Exp 2) | Anxiety | 48 | 38.77 (10.74) | 48 | 34.10 (7.41) | Paired variance unavailable; primary SE estimated using *r*=0.50 | 0.502 | 0.152 | [0.20, 0.80] |
| Hoppe 2025 (PD) | Anxiety | 29 | 21.30 (21.80) | 29 | 19.90 (21.30) | Paired variance unavailable; primary SE estimated using *r*=0.50 | 0.064 | 0.183 | [-0.30, 0.42] |
| Hoppe 2025 (HC) | Anxiety | 53 | 9.70 (17.60) | 53 | 5.20 (9.10) | Paired variance unavailable; primary SE estimated using *r*=0.50 | 0.319 | 0.140 | [0.04, 0.59] |
| Quinlan 1997 | Anxiety | 16 | 31.40 (3.20) | 16 | 33.80 (3.60) | Paired variance unavailable; primary SE estimated using *r*=0.50 | -0.687 | 0.272 | [-1.22, -0.15] |
| Razazan 2025 | Anxiety | 12 | 58.33 (12.61) | 12 | 45.25 (11.76) | Paired variance unavailable; primary SE estimated using *r*=0.50 | 1.036 | 0.350 | [0.35, 1.72] |
| Scott 2002 | Anxiety | 31 | 39.90 (11.10) | 31 | 32.40 (7.30) | Paired variance unavailable; primary SE estimated using *r*=0.50 | 0.788 | 0.204 | [0.39, 1.19] |
| Souissi 2012 | Anxiety | 12 | 1.45 (0.83) | 12 | 0.42 (0.55) | Paired variance unavailable; primary SE estimated using *r*=0.50 | 1.407 | 0.400 | [0.62, 2.19] |
| Staack 2022 | Anxiety | 56 | 7.41 (5.46) | 56 | 9.65 (5.54) | Reported within-participant adjusted estimate and SE used directly | -0.117 | 0.132 | [-0.38, 0.14] |
| Tancer 1994 (PD) | Anxiety | 10 | 55.00 (18.00) | 10 | 42.30 (9.30) | Paired variance unavailable; primary SE estimated using *r*=0.50 | 0.849 | 0.357 | [0.15, 1.55] |
| Tancer 1994 (SP) | Anxiety | 11 | 56.90 (10.60) | 11 | 43.20 (12.90) | Paired variance unavailable; primary SE estimated using *r*=0.50 | 1.116 | 0.375 | [0.38, 1.85] |
| Tancer 1994 (HC) | Anxiety | 11 | 38.40 (9.80) | 11 | 31.10 (5.40) | Paired variance unavailable; primary SE estimated using *r*=0.50 | 0.888 | 0.346 | [0.21, 1.57] |
| Distelberg 2017 | Anxiety | 28 | 6.10 (1.40) | 21 | 3.10 (1.10) | Independent-group variance | 2.304 | 0.370 | [1.58, 3.03] |
| García 2017 | Anxiety | 60 | 48.33 (4.70) | 20 | 47.27 (5.63) | Three intervention arms combined; shared control used once | 0.212 | 0.259 | [-0.29, 0.72] |

## 1.3 Supplementary Table S3

# Supplementary Table S3. Study/Comparison-Level master table

| **Study** | **Eligible intervention arms** | **Doses** | **Assessment time point(s)** | **Selected comparison** | **Reason for selection** | **Handling of shared controls, correlated comparisons, and repeated measurements** |
| --- | --- | --- | --- | --- | --- | --- |
| **Hoppe, 2025** **(34)** | Panic-disorder cohort: caffeine vs placebo; healthy-control cohort: caffeine vs placebo | 150 mg caffeine | Baseline; 30 min rest; ~40 min post-ER; ~55 min post-AACT | Caffeine vs placebo at 30 min, analyzed separately for the two cohorts | Last task-free post-dose anxiety assessment | Two independent cohorts; later repeated measures not entered |
| **Razazan, 2025** **(33)** | Placebo; caffeine; L-theanine; caffeine + L-theanine | CAF 3 mg/kg; THE 3 mg/kg; CAF+THE 3+3 mg/kg | 60 min post-dose, pre-exercise | Caffeine alone vs placebo | Direct isolated-caffeine contrast | THE and CAF+THE arms excluded; one paired effect retained |
| **Staack, 2022 (20)** | Placebo; caffeine 200 mg/day; caffeine 400 mg/day | 200 or 400 mg/day | Day 7 of each treatment condition | 200 mg/day vs placebo. | Lower eligible dose for primary pairwise analysis | Reported paired estimate used; 400-mg contrast not entered independently |
| **Yue, 2021 (26)** | Caffeinated coffee + milnacipran 75 mg/day; decaffeinated coffee + milnacipran 75 mg/day; decaffeinated coffee + milnacipran 100 mg/day | 95 mg caffeine/day | Baseline; Weeks 2, 4, 8, and 12 | Caffeinated coffee + milnacipran 75 mg/day vs decaffeinated coffee + milnacipran 75 mg/day at Week 12 | Matched milnacipran dose and final on-treatment endpoint | The 100-mg milnacipran arm is excluded because the co-intervention dose is unmatched. Earlier time points are not entered independently |
| **Chtourou, 2019 (28)** | Red Bull vs matched caffeine- and taurine-free beverage | 160 mg caffeine (plus taurine and other ingredients) | 60 min | Red Bull vs matched control at 60 min. | Only eligible matched intervention-control contrast | Single crossover comparison and single post-dose time point; no duplicate weighting |
| **Giles, 2018 (21)** | Experiment 1 and Experiment 2: caffeine vs placebo | 400 mg | Baseline, 45 and 75 min | Caffeine vs placebo at 45 min in both experiments | Latest assessment before the emotion-regulation task | Two independent cohorts; 75-min assessments not entered |
| **Liu, 2017 (27)** | Caffeine 60 mg/day + escitalopram; caffeine 120 mg/day + escitalopram; placebo + escitalopram | 60 or 120 mg/day | Baseline; Weeks 1, 2, 3, and 4 | 60 mg/day vs placebo at Week 4 | Lowest eligible caffeine dose retained under the shared-control multi-dose rule; Week 4 is the final endpoint | The 120-mg arm is not entered as a second effect, preventing repeated use of the shared placebo. Earlier weeks are not independently weighted |
| **Distelberg, 2017 (22)** | Regular caffeinated coffee vs decaffeinated coffee | 450 mg/day vs ~12 mg/day | Pretreatment Days 1 and 5; Treatment Days 3 and 5; Post-treatment Days 3 and 5 | Regular coffee vs decaffeinated coffee at Treatment Day 5 (BAI anxiety; DHP depression) | Final on-treatment anxiety assessment | One time point and one anxiety scale retained |
| **García, 2017 (30)** | Carbonated-water control; energy drinks A, B, and C | A: 149.5 mg; B: 147.2 mg; C: 155 mg caffeine | Baseline and 60 min | Combined A+B+C vs control at 60 min | Only eligible post-dose anxiety assessment | Three intervention arms combined; control entered once |
| **Souissi, 2012 (29)** | Caffeinated coffee vs placebo | 5 mg/kg caffeine | 60 min post-dose, pre-exercise | Caffeinated coffee vs placebo at 60 min | Only eligible caffeine-placebo comparison | One paired effect retained |
| **Schwartzhaupt, 2009** **(31)** | Caffeine tablets vs placebo tablets during total sleep deprivation | 450 mg Caffeine | Morning endpoint (~11 h) | Caffeine vs placebo at the morning endpoint. | Final assessment after the overnight intervention. | Only one morning crossover effect is retained; total sleep deprivation is applied to both conditions |
| **Scott, 2002 (23)** | Caffeine in orange juice vs orange-juice placebo | 5 mg/kg caffeine | Pretest; posttest after task battery | Caffeine vs placebo at pretest | Last task-free anxiety assessment | One paired effect retained; posttest excluded |
| **Quinlan, 1997 (32)** | Caffeinated and non-caffeinated tea, coffee, and water conditions | 100 mg caffeine | Baseline, 30 and 60 min | Study-defined ±caffeine contrast at 60 min | Final eligible post-dose anxiety assessment | One pooled caffeine contrast retained; 30-min measure not entered |
| **Tancer, 1994 (24)** | Caffeine vs placebo in three independent cohorts: panic disorder (PD), social phobia, and healthy controls | 480 mg | Baseline; STAI-State at 90 min; Zung at 150 min | Caffeine vs placebo at 90 min | Validated state-anxiety measure for acute response | Three independent cohorts; one effect per cohort; PD complete-pair N=10 |
| **Bernstein, 1994 (25)** | Placebo; caffeine 2.5 mg/kg; caffeine 5.0 mg/kg in matched cola | 2.5 or 5.0 mg/kg | Testing began 30 min post-dose; | 2.5mg/kg vs placebo. | Lower eligible dose selected for the primary pairwise analysis | Only one dose comparison retained |

# Note: For crossover trials, paired estimates were used when available; otherwise, the primary variance calculation assumed a within-participant correlation of *r*=0.50, with sensitivity analyses at *r*=0.25 and *r*=0.75. Only one selected comparison/time point per independent cohort and outcome was entered to avoid inappropriate weighting or double-counting.

## 1.4 Supplementary Figure S1A-D

D

C

B

A

Supplementary Figure S1A-D. Outcome-level risk-of-bias assessment using the Cochrane RoB 2 tool. (A) Traffic-light plot for depressive outcomes; (B) summary plot for depressive outcomes; (C) traffic-light plot for anxiety outcomes; and (D) summary plot for anxiety outcomes. Seven independent comparisons were assessed for depressive outcomes and 16 independent comparisons from 12 randomized controlled trials were assessed for anxiety outcomes. Summary plots show the proportion of judgments across independent comparisons. Detailed supporting rationales are provided in Supplementary Table S4.

## 1.5 Supplementary Table S4

**Supplementary Table S4. Outcome-level risk-of-bias assessment and supporting rationale using the RoB 2 tool**

| **Outcome** | **Study / target result** | **D1** | **D2** | **D3** | **D4** | **D5** | **Overall** | **Key rationale for judgment** |
| --- | --- | --- | --- | --- | --- | --- | --- | --- |
| Depression | Chtourou 2019 - POMS depression/dejection, 60 min | L | L | L | SC | L | SC | Randomization and outcome completeness were adequate. POMS was a subjective outcome and successful participant blinding was not formally verified. All POMS domains were reported. |
| Depression | Schwartzhaupt 2009 - HAMD-6 | SC | L | L | SC | SC | SC | Random-sequence generation and allocation concealment were insufficiently described. Blinding of the HAMD-6 assessor was unclear, and no accessible prespecified protocol identified the selected depression result. |
| Depression | Souissi 2012 - POMS depression/dejection, 60 min | SC | L | L | SC | L | SC | Randomized order was reported without details of sequence generation or concealment. Participant blinding was insufficiently described for a subjective POMS outcome; all POMS domains were reported. |
| Depression | Staack 2022 - BDI, Day 7 | SC | L | SC | L | L | SC | Randomization procedures were incompletely described. Of 81 randomized participants, 56 were analyzed, with several withdrawals insufficiently explained. Outcome measurement and reporting were otherwise well specified. |
| Depression | Distelberg 2017 - DHP depression, Treatment Day 5 | SC | L | SC | L | L | SC | Randomization methods were incompletely described. Effective sample sizes varied across outcome models and reasons for missing observations were not fully reported. Multiple depression measures and time points were reported. |
| Depression | Liu 2017 - HDRS-17, Week 4 | SC | L | SC | L | SC | SC | Allocation procedures were incompletely described. Participant-flow numbers were internally inconsistent, and several depression scales/time points were available without a verifiable prespecified analysis plan. |
| Depression | Yue 2021 - HAMD-24, Week 12 | SC | L | SC | SC | L | SC | Allocation concealment was not reported and participant accounting was inconsistent. Some withdrawals may have been related to treatment response, and assessor blinding for HAMD-24 was not stated. All scheduled HAMD-24 assessments were reported. |
| Anxiety | Bernstein 1994 - STAI-State, 2.5 mg/kg | SC | L | L | L | L | SC | Treatment order was randomized, but sequence generation and concealment were not described. Blinding and outcome completeness were adequate, and the relevant anxiety measures were reported. |
| Anxiety | Chtourou 2019 - POMS anxiety/tension, 60 min | L | L | L | SC | L | SC | Randomization was adequately described. POMS was subjective and successful participant blinding was not formally verified; all POMS domains were reported. |
| Anxiety | Giles 2018 (Exp 1) - STAI-State, 45 min | SC | L | L | SC | L | SC | Treatment order was counterbalanced but sequence generation/concealment were incompletely described. Participant treatment guesses were not assessed for this subjective outcome; prespecified STAI time points were reported. |
| Anxiety | Giles 2018 (Exp 2) - STAI-State, 45 min | SC | L | L | SC | L | SC | Same design-related concerns as Experiment 1; outcome data were complete and the prespecified STAI assessments were reported. |
| Anxiety | Hoppe 2025 (PD) - NRS anxiety, 30 min | L | L | L | L | L | L | Independent block randomization, double blinding, a prespecified primary anxiety outcome, and comprehensive reporting supported low risk across domains. |
| Anxiety | Hoppe 2025 (HC) - NRS anxiety, 30 min | L | L | L | L | L | L | The same well-described randomization, blinding, outcome measurement, and prespecified reporting procedures applied to the healthy-control cohort. |
| Anxiety | Quinlan 1997 - STAI-State, 60 min | L | SC | L | SC | L | SC | Latin-square crossover allocation was reported. A small number of treatment-allocation deviations occurred, and participants showed some ability to distinguish caffeinated conditions; both STAI time points were reported. |
| Anxiety | Razazan 2025 - STAI-State, 60 min | L | L | L | L | L | L | Independent supplement coding/randomization, strict double blinding, identical capsules, complete outcome data, and comprehensive reporting supported low risk. |
| Anxiety | Scott 2002 - STAI-State pretest | SC | L | L | SC | L | SC | Randomization procedures were incompletely described. The timing of the selected STAI assessment was inconsistently reported between the Methods and other sections of the article. |
| Anxiety | Souissi 2012 - POMS anxiety/tension, 60 min | SC | L | L | SC | L | SC | Randomized order was reported without sequence-generation details. Participant blinding was insufficiently described for the subjective POMS outcome; all mood domains were reported. |
| Anxiety | Staack 2022 - BAI, Day 7 | SC | L | SC | L | L | SC | Randomization procedures were incompletely described. Attrition was substantial, with 56 of 81 randomized participants analyzed and several withdrawals unexplained. |
| Anxiety | Tancer 1994 (HC) - STAI-State, 90 min | SC | L | L | SC | SC | SC | Treatment sequence was not clearly randomized. High-dose caffeine could have compromised participant blinding for a self-reported outcome, and multiple anxiety measures/time points were available without a prespecified protocol. |
| Anxiety | Tancer 1994 (PD) - STAI-State, 90 min | SC | L | SC | SC | SC | SC | Same concerns as the healthy-control cohort, with additional incomplete paired data because one panic-disorder participant received caffeine only. |
| Anxiety | Tancer 1994 (SP) - STAI-State, 90 min | SC | L | L | SC | SC | SC | Treatment sequence and maintenance of participant blinding were uncertain; multiple anxiety measures/time points were reported without an accessible prespecified protocol. |
| Anxiety | Distelberg 2017 - BAI, Treatment Day 5 | SC | L | SC | L | L | SC | Randomization methods were incompletely described. The effective sample size for BAI analyses was lower than the randomized sample without complete explanation; multiple anxiety measures and time points were nevertheless reported. |
| Anxiety | Garcia 2017 - STAI-State, 60 min | SC | L | L | SC | L | SC | Randomization was reported without sequence-generation or concealment details. Investigators were blinded, but participants could potentially identify energy-drink conditions by taste; STAI analyses were comprehensively reported. |

**Abbreviations and domain definitions:** D1, bias arising from the randomization process; D2, bias due to deviations from intended interventions; D3, bias due to missing outcome data; D4, bias in measurement of the outcome; D5, bias in selection of the reported result; L, low risk; SC, some concerns; BAI = Beck Anxiety Inventory; BDI = Beck Depression Inventory; DHP = Duke Health Profile; HAMD = Hamilton Depression Rating Scale; NRS = Numerical Rating Scale; POMS = Profile of Mood States; STAI = State-Trait Anxiety Inventory.

## 1.6 Supplementary Figure S2

**Supplementary Figure S2**. Meta-analysis of acute depressive mood responses in non-psychiatric populations.

## 1.7 Supplementary Figure S3

**Supplementary Figure S3**. Meta-analysis of acute anxiety responses in non-psychiatric populations.

## 1.8 Supplementary Figure S4

**Supplementary Figure S4.** Exploratory dose-stratified analysis of depressive symptoms

## 1.9 Supplementary Figure S5

**Supplementary Figure S5.** Exploratory subgroup analysis of depressive symptoms by assessment modality.

## 1.10 Supplementary Table S5

**Supplementary Table S5. Sensitivity analyses for crossover trials under different within-participant**

**correlation assumptions**

| **Outcome** | **Correlation assumption** | **Hedges’ g** | **95% CI** | ***P*** | ***τ²*** |
| --- | --- | --- | --- | --- | --- |
| Depression | *r*=0.25 | -0.496 | [−1.334, 0.342] | 0.198 | 0.745 |
| Depression | *r*=0.50 | -0.493 | [−1.327, 0.340] | 0.198 | 0.738 |
| Depression | *r*=0.75 | -0.490 | [-1.320, 0.339] | 0.198 | 0.731 |
| Anxiety | r=0.25 | 0.503 | [0.115, 0.891] | 0.014 | 0.413 |
| Anxiety | r=0.50 | 0.505 | [0.118, 0.892] | 0.014 | 0.429 |
| Anxiety | r=0.75 | 0.508 | [0.121, 0.894] | 0.013 | 0.447 |

## 1.11 Supplementary Table S6

**Supplementary Table S6. Sensitivity analysis restricted to isolated caffeine contrasts**

| **Outcome** | **Independent comparisons** | **Hedges' g** | **95% CI** | ***P*** | **τ²** | ***I*² (%)** | **Q (P)** |
| --- | --- | --- | --- | --- | --- | --- | --- |
| Depression | 3 | −0.341 | [−1.598, 0.915] | 0.363 | 0.215 | 83.38 | 12.87 (0.002) |
| Anxiety | 11 | 0.471 | [0.206, 0.736] | 0.003 | 0.099 | 72.06 | 33.93 (<0.001) |

*Note:* An isolated caffeine contrast was defined as a comparison in which caffeine was the differential active component between intervention and control conditions. Matched vehicles and background treatments common to both groups were permitted; coffee, tea, energy drinks, and other multi-component caffeine-containing products were excluded.

## 1.12 Supplementary Table S7

**Supplementary Table S7. Leave-one-out sensitivity analyses for depressive and anxiety outcomes**

| **Outcome** | **Omitted comparison** | **Hedges' g** | **95% CI** | ***P*** |
| --- | --- | --- | --- | --- |
| Depression | Chtourou 2019 | -0.523 | [-1.555, 0.508] | 0.249 |
| Depression | Schwartzhaupt 2009 | -0.511 | [-1.548, 0.526] | 0.261 |
| Depression | Souissi 2012 | -0.574 | [-1.581, 0.432] | 0.202 |
| Depression | Staack 2022 | -0.606 | [-1.594, 0.382] | 0.175 |
| Depression | Distelberg 2017 | -0.625 | [-1.578, 0.329] | 0.153 |
| Depression | Liu 2017 | -0.432 | [-1.451, 0.587] | 0.325 |
| Depression | Yue 2021 | -0.176 | [-0.619, 0.266] | 0.353 |
| **Depression** | **Primary analysis (no omission)** | **-0.493** | **[-1.327, 0.340]** | **0.198** |
| Anxiety | Bernstein 1994 | 0.529 | [0.114, 0.944] | 0.016 |
| Anxiety | Chtourou 2019 | 0.573 | [0.192, 0.954] | 0.006 |
| Anxiety | Giles 2018 (Exp 1) | 0.512 | [0.093, 0.931] | 0.020 |
| Anxiety | Giles 2018 (Exp 2) | 0.509 | [0.090, 0.928] | 0.021 |
| Anxiety | Hoppe 2025 (PD) | 0.539 | [0.127, 0.951] | 0.014 |
| Anxiety | Hoppe 2025 (HC) | 0.522 | [0.105, 0.940] | 0.018 |
| Anxiety | Quinlan 1997 | 0.577 | [0.204, 0.950] | 0.005 |
| Anxiety | Razazan 2025 | 0.474 | [0.066, 0.882] | 0.026 |
| Anxiety | Scott 2002 | 0.488 | [0.072, 0.903] | 0.025 |
| Anxiety | Souissi 2012 | 0.453 | [0.059, 0.847] | 0.027 |
| Anxiety | Staack 2022 | 0.552 | [0.147, 0.958] | 0.011 |
| Anxiety | Tancer 1994 (HC) | 0.484 | [0.072, 0.896] | 0.025 |
| Anxiety | Tancer 1994 (PD) | 0.487 | [0.074, 0.899] | 0.024 |
| Anxiety | Tancer 1994 (SP) | 0.470 | [0.065, 0.876] | 0.026 |
| Anxiety | Distelberg 2017 | 0.384 | [0.063, 0.705] | 0.022 |
| Anxiety | Garcia 2017 | 0.528 | [0.113, 0.942] | 0.016 |
| **Anxiety** | **Primary analysis (no omission)** | **0.505** | **[0.118, 0.892]** | **0.014** |

Note: Effect sizes are expressed as Hedges' g. Leave-one-out analyses used random-effects models with restricted maximum likelihood (REML) estimation and Hartung-Knapp adjustment. Analyses were conducted at the independent-comparison level; independent experiments or cohorts from the same study were treated as separate comparisons where applicable. Primary-analysis rows are shown for reference.

## 1.13 Supplementary Figure S6

**Supplementary Figure S6**. Funnel plot for depressive outcomes.

## 1.14 Supplementary Figure S7

**Supplementary Figure S7**. Funnel plot for anxiety outcomes.

## 1.15 Supplementary Table S8

**Supplementary Table S8: Table of the GRADE Assessment**

**Author(s):** Ailing Zhang; Yudan Ni; Zhongyu Zhang

**Question:** Figure of the GRADE Assessment

**Setting:** Community and clinical settings

**Bibliography:**

| **Certainty assessment** | | | | | | | **№ of patients** | | **Effect** | | **Certainty** | **Importance** |
| --- | --- | --- | --- | --- | --- | --- | --- | --- | --- | --- | --- | --- |
| **№ of studies** | **Study design** | **Risk of bias** | **Inconsistency** | **Indirectness** | **Imprecision** | **Other considerations** | **[Caffeine]** | **[Placebo]** | **Relative (95% CI)** | **Absolute (95% CI)** |  |  |
| **Depressive symptoms** | | | | | | | | | | | | |
| 7 | randomised trials | serious^a^ | serious^b^ | serious^c^ | serious^d^ | none | 206 | 199 | - | SMD **0.49 SD lower** (1.33 lower to 0.34 higher) | ⨁◯◯◯ Very low^a,b,c,d^ | CRITICAL |
| **Anxiety symptoms** | | | | | | | | | | | | |
| 12 | randomised trials | serious^e^ | serious^f^ | serious^g^ | serious^h^ | publication bias strongly suspected^i^ | 464 | 417 | - | SMD **0.51 SD higher** (0.12 higher to 0.89 higher) | ⨁◯◯◯ Very low^e,f,g,h,i^ | CRITICAL |

**CI:** confidence interval; **SMD:** standardised mean difference

#### Explanations

a. All seven comparisons were judged as having some concerns overall, mainly due to limitations in the randomization process, with additional concerns regarding missing outcome data, outcome measurement, and selective reporting. No comparison was rated at high risk of bias; therefore, the evidence was downgraded by one level.

b. Effect estimates varied substantially in magnitude and direction across the seven comparisons, with considerable statistical heterogeneity (I² = 92.9%, τ² = 0.74) and a wide 95% prediction interval (−2.87 to 1.88).

c. Downgraded one level because of differences in study populations, caffeine exposure patterns and duration, and depressive outcome measures, which limited the direct applicability of the pooled estimate to a single clinically coherent setting.

d. The 95% CI was wide (SMD = −0.49, 95% CI [−1.33, 0.34]) and included effects ranging from a potentially substantial reduction to a possible increase in depressive symptom scores. No common minimally important difference was available across the different outcome scales.

e. Thirteen of 16 anxiety comparisons were judged as having some concerns overall, mainly related to the randomization process and outcome measurement. Although no comparison was rated at high risk of bias, the prevalence of these concerns warranted one-level downgrading.

f. Effect estimates varied substantially across comparisons, with considerable heterogeneity (I² = 90.3%, τ² = 0.43) and a wide 95% prediction interval (−0.95 to 1.96). Heterogeneity persisted within clinically more comparable subgroups and was not fully explained by subgroup analyses.

g. Downgraded one level because of differences in participant populations, caffeine exposure patterns and duration, and anxiety outcome measures, which limited the direct applicability of the pooled estimate to a single clinically coherent setting.

h. The 95% CI (0.12 to 0.89) encompassed effects ranging from a small to a considerably larger increase in anxiety symptoms. No common minimally important difference could be defined across the different anxiety scales, limiting certainty about the clinical magnitude of the effect.

i. Funnel-plot asymmetry and a significant Egger’s test (z = 2.74, p = 0.0062) suggested small-study effects. Although heterogeneity may have contributed to the asymmetry, publication bias was considered sufficiently plausible to warrant one-level downgrading.
